# Anti‐Corrosive Covalent Iodo‐Thiadiazole Catalyst Enables Aqueous Zn─S Batteries with High Coulombic Efficiency

**DOI:** 10.1002/adma.202508570

**Published:** 2025-08-18

**Authors:** Jiahao Liu, Yujie Chen, Han Wu, Chao Ye, Shi‐Zhang Qiao

**Affiliations:** ^1^ School of Chemical Engineering The University of Adelaide Adelaide SA 5005 Australia

**Keywords:** aqueous Zn─S batteries, high coulombic efficiency, redox mediators, Zn anode corrosion, Zn─S redox intermediates

## Abstract

Aqueous zinc‐sulfur batteries (AZSBs) hold great promise for large‐scale energy storage but suffer from low Coulombic efficiency (CE) due to sluggish Zn─S redox kinetics and severe Zn anode corrosion, limiting their cycling life and practical applicability. Although state‐of‐the‐art iodine‐based redox mediators can accelerate cathode kinetics, they typically result in the formation of free I_3_^−^ species, which exacerbate Zn corrosion. Here, we report a covalent iodo–thiadiazole redox mediator (CIM) as an anti‐corrosive and highly efficient catalyst to achieve high CE in AZSBs. The covalently anchored iodine in CIM effectively suppresses free I_3_^−^ formation and mitigates Zn corrosion. More importantly, dynamic electronic restructuring from C5═N to C5─N bonds (C5: 5th‐position carbon in the thiadiazole ring) promotes Zn–S redox kinetics through a σ‐coordination electron pathway facilitated by the Zn─I─C5 bond. As a result, CIM‐based coin cells achieve an average CE of 99.56% and a capacity of 344 mAh g^−1^ after 700 cycles at 8 C. The CIM‐based pouch cell demonstrates a high capacity of 1398 mAh g^−1^ after 120 cycles at 0.8 C. This work presents a practical design strategy for iodine‐based catalysts, enabling next‐generation aqueous metal‐sulfur batteries with enhanced durability and performance.

## Graphical abstract

In aqueous Zn–S batteries, a covalently bonded iodo–thiadiazole redox mediator suppresses parasitic I_3_^−^‐mediated Zn corrosion and enhances Zn–S redox kinetics, enabling pouch cell with a high capacity of 1398 mAh g^−1^ after 120 cycles. This innovative catalyst design is a leap toward practical and grid‐scale aqueous Zn–S batteries.

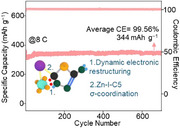

## 1 Introduction

Aqueous Zn–S batteries (AZSBs) have emerged as a promising candidate for grid‐scale energy storage, owing to their low cost of approximately 52 Ah $^−1^, high theoretical capacity of 1675 mAh g^−1^, and inherent safety.^[^ ^1^ ^]^ However, their practical implementation is hindered by poor Coulombic efficiency (CE), resulting in approximately 70% capacity retention after 30 cycles.^[^ ^2^ ^]^ This issue primarily arises from sluggish solid‐solid Zn–S redox kinetics, resulting in low sulfur utilization and the accumulation of inactive “dead S” and “dead ZnS.”^[^ ^3^ ^]^ To improve Zn–S redox kinetics, iodine‐based redox mediators have been widely investigated.^[^ ^4^ ^]^ Such mediators leverage the I^−^/I_3_^−^ redox couple to catalyze the conversion from S_8_ to ZnS during discharge, achieving an improved average CE of 98% and extending cycle life to ≈300 cycles. However, even if the average CE reaches 98%, only 40% capacity can be retained after 300 cycles. This is significantly lower than the requirement of 80% capacity retention over 500 cycles for practical energy storage systems, corresponding to the threshold CE of 99.5%.^[^ ^5^ ^]^

The primary challenge in achieving high CE lies in addressing the I_3_^−^‐mediated Zn anode corrosion.^[^ ^6^ ^]^ This corrosion includes an accumulation of passivating by‐products such as Zn(OH)_2_,^[^ ^7^ ^]^ and a parasitic oxygen evolution reaction (OER) mediated by I_3_^−^: 4I_3_^−^ + 6Zn(OH)_2_ → 6ZnI_2_ + 3O_2_ + 6H_2_O.^[^ ^8^ ^]^ Previous efforts to mitigate the I_3_^−^‐mediated corrosion have been proposed in an aqueous Zn‐I_2_ battery. For instance, Ren et al.^[^ ^9^ ^]^ introduced an organic fucoidan corrosion inhibitor to form an artificial SEI protective layer on Zn anode. However, these protective layers are insufficient to block highly corrosive I_3_^−^ during long cycling, leading to continuous iodine‐species depletion through parasitic reactions and ultimately resulting in reduced CE. Another strategy is employing iodine‐based ionic complex such as thiourea–iodide (TUI) to anchor iodine species and suppress their dissolution. Such ionic complexes, however, tend to dissociate into TU^+^ and I^−^ in aqueous electrolytes, resulting in the formation of corrosive I_3_^−^.^[^ ^10^ ^]^ Therefore, the development of an iodine‐based catalyst capable of minimizing iodine species dissolution is essential to simultaneously suppress Zn anode corrosion and accelerate Zn–S redox kinetics, thereby enhancing CE in AZSBs.

Herein, we develop an anti‐corrosive covalent iodo‐thiadiazole redox mediator (CIM).^[^ ^11^ ^]^ Specifically, the covalent C5–I bond, where C5 refers to the fifth carbon position on the thiazole ring, in the CIM structure anchors iodine species, effectively suppressing the I_3_^−^‐mediated anode corrosion. This effect is demonstrated by the in situ gas chromatography‐mass spectrometry (GC‐MS) and in situ ultraviolet‐visible (UV–Vis) spectroscopy. Synchrotron infrared micro‐spectroscopy (IRM) and solid‐state nuclear magnetic resonance (SSNMR) further confirm that the dynamic electronic restructuring effect of the C5═N→C5─N transition in the CIM.^[^ ^11^, ^12^ ^]^ This facilitates continuous electron donation to Zn–S redox intermediates via the Zn─I─C5 bond, as revealed by in situ synchrotron IRM and synchrotron near‐edge X‐ray absorption fine structure (NEXAFS). Due to this synergistic mechanism, the CIM‐based pouch cell delivers a capacity of 1398 mAh g^−1^ after 120 cycles at 0.8 C. These findings highlight CIM as an advanced redox mediator and offer a promising pathway toward the realization of practical aqueous metal‐sulfur batteries.

## 2 Results and Discussion

### 2.1 Synthesis and Structural Characterization of CIM

**Figure 1:**
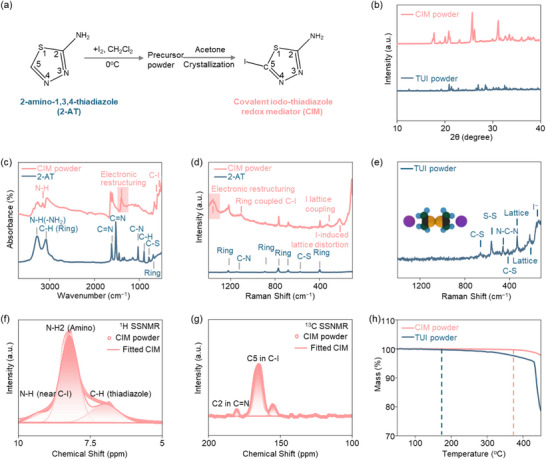
a) Synthesis of CIM via controlled iodination. b) XRD patterns of CIM and TUI powders. c,d) Synchrotron IRM and Raman spectra of CIM powder, exhibiting an amplified electronic restructuring effect. The standard 2‐AT spectrum is obtained from the SpectraBase database (ID: 4PgA7hilt80). e) Raman spectra of the TUI powder. f,g) ^1^H and ^13^C SSNMR spectra of CIM powder. h) TGA profiles of CIM and TUI powders.

The CIM and TUI ([(NH_2_)_2_C─S─S─C(NH_2_)_2_]^2+^∙2I^−^ as a control sample) were synthesized via controlled iodination of the commercial precursors 2‐amino‐1,3,4‐thiadiazole (2‐AT) and thiourea (TU), respectively, based on a modified Biesiada protocol (**Figure** **1**a).^[^ ^11^ ^]^ Specifically, CIM is synthesized by mixing 2‐AT and I_2_ in a 1:1 molar ratio in dichloromethane, followed by stirring at 500 rpm and 0 °C for 3 h. The product is then crystallized in acetone and dried at 60 °C for 12 h. The selective electrophilic iodination occurs at the C5 position due to its high electron density in the thiadiazole ring.^[^ ^13^ ^]^ The TU undergoes a similar iodination process but crystallizes directly from dichloromethane (Figure S1, Supporting Information). The distinct color change from white to brown implies iodination of 2‐AT and TU (Figure S2, Supporting Information). X‐ray diffraction (XRD) analysis in Figure 1b and Figure S3 (Supporting Information) reveals the structure evolution from the precursors to CIM and TUI, indicating the significant molecular reorganization.^[^ ^14^, ^15^ ^]^

To further investigate the structure of CIM and TUI, synchrotron IRM was performed to analyze functional group features (Figure 1c; Tables S1 and S2, with peak analysis provided in the Supporting Information). For the 2‐AT, IRM reveals a partial decreased bond order from C5═N to C5─N in the thiadiazole ring, as evidenced by the peak at 1448 cm^−1^, which represents an intermediate state between the C═N and the C─N with characteristic stretching frequencies at 1500‐1650 and 1250‐1350 cm^−1^, respectively, indicating a weak electronic restructuring effect on the C5 (C5═N→C5─N). Upon iodination to CIM, this electronic restructuring effect is significantly amplified as evidenced by a redshift of the peak at 1448 to 1401 cm^−1^. This is attributed to the electron withdrawing of the covalent C5─I bond identified at 549 and 596 cm^−1^. This electronic restructuring effect is further evidenced by another split peak of N─H at 3159 cm^−1^. Moreover, the C2═N peak undergoes a blueshift from 1527 to 1599 cm^−1^ due to electron localization, resulting from the formation of the covalent C5─I bond. Compared with the Raman spectra of 2‐AT, there are characteristic vibrational modes in CIM powder at 1368 cm^−1^ (C5═N→C5─N), 329 cm^−1^ (C5─I stretching), and low‐frequency lattice vibrations at 227 and 110 cm^−1^ (covalent C5─I bond), as shown in Figure 1d,e and Tables S3–S5 (with peak analysis provided in the Supporting Information). In contrast, the Raman spectrum of TUI exhibits characteristic vibrational modes including S─S stretching (568 cm^−1^), C─S stretching (655 cm^−1^), N─C─N bending (458 cm^−1^), and I^−^ lattice vibration at 568, 655, 458, and 157 cm^−1^, respectively. These features confirm the ionic structure of the TUI.

To probe the local structure and molecular environment of CIM, SSNMR tests were performed to characterize the hydrogen and carbon chemical environments in both CIM and TUI, as shown in Figure 1f,g and Tables S6 and S7 (with peak analysis provided in the Supporting Information). The ^1^H spectrum of CIM reveals that the peaks at 9.2, 8.2, and 6.9 ppm represent the H environments of N4─H, remote amino group, and C─H within the thiadiazole ring, respectively, aligning well with the vibrational data from synchrotron IRM and Raman spectroscopy. The ^13^C SSNMR spectrum reveals three distinct carbon environments in CIM: a highly deshielded C2═N carbon (180.3 ppm) in the thiadiazole ring, a strong C5─I bonded carbon (165.3 ppm) with partial shielding compensation, and a mixed C5─N/C5═N configuration (155.5 ppm). In contrast, the ^1^H SSNMR spectrum of TUI (Figure S4, Supporting Information) reveals four distinct hydrogen environments influenced by the electronic effects as shown in detail in Table S8 (Supporting Information). In addition, thermal gravimetric analysis (TGA) results show that the CIM decomposes at ≈370 °C due to its covalent C5─I bond, suggesting good thermal stability (Figure 1h). In contrast, TUI decomposes at a significantly lower temperature of 170 °C and exhibits rapid mass loss above 450 °C. Raman spectroscopy (Figure S5, Supporting Information) confirms that this mass loss originates from iodine species, likely due to the weaker binding affinity of TUI for iodine species.^[^ ^16^ ^]^ These results suggest the stable covalent C5─I bond as an anchor for iodine species to eliminate I_3_^−^‐mediated Zn anode corrosion, and the electronic restructuring on C5 as a potential electron donor to catalyze the cathode Zn–S redox on cathode side. To assess the air stability of CIM and TUI, we have monitored both samples under ambient conditions and present our findings in Figures S6a–c (Supporting Information, photographs were taken after 3 months, and UV–Vis spectra were recorded after 10 days). TUI exhibits iodine volatility, as evidenced by the purple discoloration of the container and the UV–Vis detection of I_3_^−^ species. In contrast, CIM demonstrates much better air stability, only minimal changes are detectable in UV–Vis spectra over the monitoring period.

### 2.2 Anti‐Corrosion Capability of CIM on Zn Anodes

**Figure 2:**
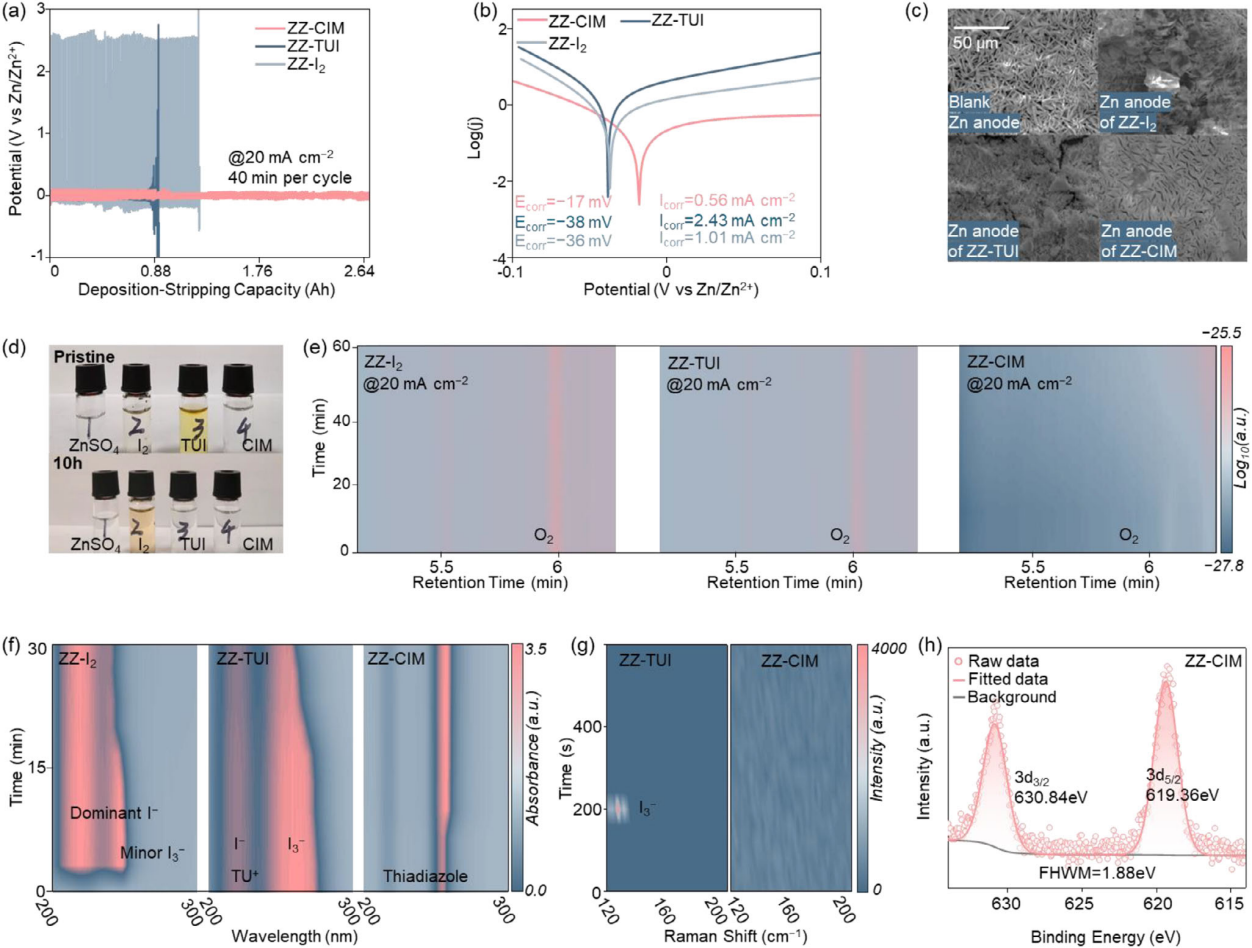
a) Long‐term Zn deposition/stripping and b) Tafel plots with ZZ‐I_2_, ZZ‐TUI, and ZZ‐CIM. c) SEM images of Zn anodes with electrolytes of 2 m ZnSO_4_, ZZ‐I_2_, ZZ‐TUI, and ZZ‐CIM. d) Color evolution of the four electrolytes in 10 h. e) In situ GC‐MS results of I_3_^−^‐mediated OER with ZZ‐I_2_, ZZ‐TUI, and ZZ‐CIM. f) In situ UV–Vis spectra with ZZ‐I_2_, ZZ‐TUI, and ZZ‐CIM. g) In situ Raman spectra with ZZ‐TUI and ZZ‐CIM. h) XPS analysis of the I 3d on Zn anodes with ZZ‐CIM.

The anti‐corrosion capabilities of various catalysts against the two‐step I_3_^−^‐mediated corrosion on Zn anode were systematically evaluated. Zn||Zn symmetric batteries were assembled using the following electrolytes: 1 wt. % I_2_, TUI and CIM in 2 m ZnSO_4_ (ZZ‐Blank) denoted as ZZ‐I_2_, ZZ‐TUI and ZZ‐CIM, respectively. Their corresponding ionic conductivity values at 20 °C, measured via a standard solution conductivity detection method, are shown in Figure S7 (Supporting Information). The ZZ‐CIM exhibits the highest ionic conductivity of 32.3 mS cm^−1^, surpassing those of ZZ‐Blank (30.5 mS cm^−1^), ZZ‐I_2_ (29.3 mS cm^−1^), and ZZ‐TUI (29.6 mS cm^−1^). As shown in **Figure** **2**a, under rigorous Zn deposition/stripping conditions of 20 mA cm^−2^ and 40 min per cycle, ZZ‐CIM sustained stable cycling for 2.64 Ah with optimized polarization, outperforming 1.28 and 0.88 Ah with ZZ‐I_2_ and ZZ‐TUI, respectively (Figure S8, Supporting Information). The failure mechanism of ZZ‐CIM and ZZ‐TUI is assigned to the open‐circuit failures, resulting from the accumulation of passivating by‐products Zn(OH)_2_, which is the initial corrosion product in I_3_^−^‐mediated Zn corrosion.^[^ ^7^, ^8^, ^9^ ^]^ In Figure 2b, Tafel analysis quantified the anti‐corrosion capabilities of the three electrolytes, in which ZZ‐CIM effectively suppressed the Zn corrosion with the highest *E_corr_* of −17 mV and lowest *I_corr_* of 0.56 mA cm^−2^, outperforming the ZZ‐I_2_ (*E_corr_* = −36 mV; *I_corr_* = 1.01 mA cm^−2^) and the ZZ‐TUI (*E_corr_* = −38 mV; *I_corr_* = 2.43 mA cm^−2^). This trend is further confirmed by scanning electron microscopy (SEM) images. As shown in Figure 2c, severe passivating by‐products of Zn(OH)_2_ were observed on anode surfaces in ZZ‐I_2_ and ZZ‐TUI, while clean and flat anode surfaces were observed with ZZ‐CIM.^[^ ^7^, ^8^, ^9^ ^]^ These further demonstrate the anti‐corrosive property of CIM. In addition, the degree of corrosion can be correlated with free I_3_^−^ concentration (Figure 2d).^[^ ^8b^ ^]^ The ZZ‐CIM remained colorless due to iodine confinement by the covalent C5–I bond. In contrast, the color of ZZ‐TUI switched from yellow to colorless, resulting from the decomposition of I_3_^−^. The color of ZZ‐I_2_ gradually darkened due to the gradual release of I_3_^−^.

The I_3_^−^‐mediated OER in Zn anode corrosion was further studied by in situ GC‐MS analysis (Figure 2e). The oxygen amounts significantly increased in ZZ‐I_2_ and ZZ‐TUI. This is because the free I_3_^−^ reacted with the passivating by‐products Zn(OH)_2_ and drove OER. In contrast, ZZ‐CIM suppressed the formation of free I_3_^−^ species and I_3_^−^‐mediated OER due to the stable covalent C5–I bond in CIM. The pH analysis for the three electrolytes also supports the I_3_^−^‐mediated OER tendency (Figure S9, Supporting Information). The ZZ‐CIM exhibited the highest pH value of 4.43. In contrast, pH values of 3.65 and 2.61 were tested in the ZZ‐I_2_ and ZZ‐TUI electrolyte. These pH trends can be explained by the hydrolysis of I_3_^−^ to generate acidic hydroiodic acid (HI).^[^ ^8b^ ^]^

In situ UV–Vis spectroscopy, in situ Raman spectroscopy, and X‐ray photoelectron spectroscopy (XPS) were employed to elucidate the anti‐corrosion mechanism of CIM, as shown in Figure 2f–h and Figure S10 (Supporting Information). In situ UV–Vis spectra of ZZ‐Blank and ZZ‐CIM exhibited no I_3_^−^ signals. In contrast, the ZZ‐TUI exhibited distinct peaks at ≈226 and ≈285 nm, corresponding to I^−^ and I_3_^−^, respectively, demonstrating significant evolution of the I^−^/I_3_^−^ redox couple during cycling. Similarly, ZZ‐I_2_ exhibited a dominant I^−^ peak with a minor I_3_^−^, resulting from the relatively low solubility of I_2_. These observations are further confirmed by the in situ Raman spectra, where I_3_^−^ species at ≈128 cm^−1^ were observed in ZZ‐TUI. Noted that I^−^ signal may not be detected from Raman spectra. Additionally, I 3d XPS analysis on the Zn anode with ZZ‐CIM system reveals a predominant iodine valent state of −1, compared to the mixed iodine valent states of −1 and 0 with ZZ‐TUI (Figure S11, Supporting Information). In summary, ZZ‐CIM demonstrates significant anti‐corrosion capacity by effectively eliminating the I_3_^−^‐mediated anode corrosion through its covalent C5─I bond, enabling high CEs on the Zn anode side. In contrast, ZZ‐I_2_ and ZZ‐TUI suffer from severe I_3_^−^‐mediated Zn anode corrosion.

### 2.3 CIM Accelerates Zn–S Redox Kinetics on Sulfur Cathode

**Figure 3:**
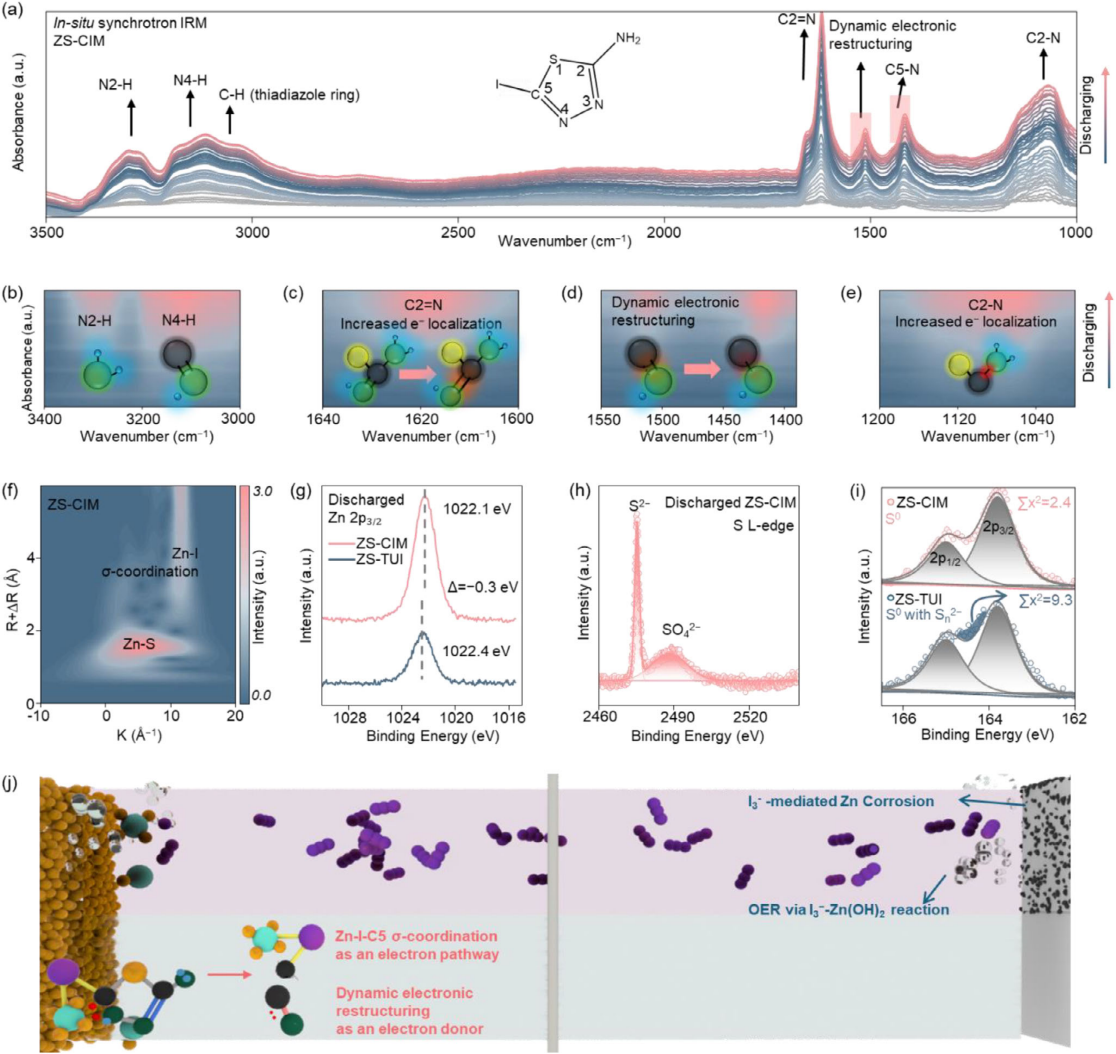
a) In situ synchrotron IRM analysis of ZS‐CIM with b‐e) contour plots (detailed spectral analysis in Table S9, Supporting Information). f) WT‐XAFS contour plots of ZS‐CIM. g) XPS analysis of the Zn 2p region in ZS‐CIM and ZS‐TUI. h) NEXAFS spectrum of sulfur of the charged cathode. i) XPS spectrum after complete charging in ZS‐CIM and ZS‐TUI. j) The synergistic mechanism of CIM, including anti‐corrosion and enhanced cathode kinetics.

To investigate the mechanism of CIM catalyzing the cathode Zn–S redox kinetics, systematic characterizations were conducted with three battery configurations: Zn–S batteries with additives of I_2_, TUI, and CIM are denoted as ZS‐I_2_, ZS‐TUI, and ZS‐CIM, respectively. The Raman spectrum collected at open‐circuit voltage (Figure S12, Supporting Information) reveals that CIM facilitates the activation of S_8_ to an intermediate state S_8_^*^, thereby accelerating the Zn–S redox kinetics. During discharge, in situ synchrotron IRM analysis demonstrates that the dynamic electronic restructuring effect of CIM facilitated the charge donation to Zn–S redox intermediates, as shown in **Figure** **3**a–e and Table S9 (Supporting Information). A redshift from 1420 to 1410 cm^−1^ demonstrates a significant electronic restructuring from C5═N to C5─N, accompanied by an intensified N4─H split peak at 3115 cm^−1^ (Figure 3b). As shown in Figure 3d, a peak emerging at 1509 cm^−1^ corresponds to a metastable intermediate originated from the dynamic restructuring from C5═N to C5─N bond, demonstrating the electron‐donating role of CIM. This dynamic process induces intensified C2═N and C2─N vibrations at 1635 and 1069 cm^−1^, as shown in Figure 3c–e. This disruption of the conjugated π‐system in the thiadiazole ring increased electron localization in the C2═N bond.^[^ ^17^ ^]^ In contrast, as shown in Figure S13 and Table S10 (Supporting Information), in situ IRM results reveal that TUI accelerates Zn–S redox through the I^−^/I_3_^−^ redox, similar to the mechanism with ZS‐I_2_.

The synchrotron X‐ray absorption fine structure‐based wavelet transform analysis (WT‐XAFS) results further demonstrate the interaction between the CIM and Zn–S redox intermediates via distinct σ‐coordination of the Zn─I─C5 validated by the scattering signals at high *k*‐values (10 Å^−1^) and *R+ΔR* regions (> 2.5 Å) (Figure 3f and Figure S14, Supporting Information). In contrast, in ZS‐TUI, only Zn─S bond was detected in Zn–S redox intermediates, as shown in Figure S15 (Supporting Information). Furthermore, Zn 2p_3/2_ binding energy of 1022.1 eV in ZS‐CIM compared with that of 1022.4 eV in ZS‐TUI suggests that the Zn─I─C5 σ‐coordination reduces the electron density on Zn during discharge processes, as shown in Figure 3g. This optimizes the energy barrier for ZnS formation by regulating the binding strength of the key Zn–S redox intermediates.

The increased sulfur utilization in ZS‐CIM via enhanced Zn–S redox kinetics was further demonstrated by the synchrotron NEXAFS spectra. As shown in Figure 3h, the dominant S^2−^ signal and the absence of the S_8_ signal indicate a complete conversion from S_8_ to ZnS in ZS‐CIM after discharge. During the charge process, the conversion from ZnS to S_8_ occurs at a low onset potential at 1.1 V (Figure S16, Supporting Information), and ZnS is completely converted back to S_8_ after charging in ZS‐CIM. This is confirmed by the fitting results of standard S_8_ in XPS analysis of the S 2p region in Figure 3i, with a small ∑χ^2^ value of 2.4. This observation is further supported by in situ Raman spectra, showing a complete conversion between S_8_ and ZnS, as shown in Figure S17 (Supporting Information). In contrast, the conversion is incomplete in ZS‐TUI, as indicated by the co‐existence of S_8_ and S_n_^2−^ species. These results demonstrate that the CIM significantly improves cathode Zn–S redox kinetics through the dynamic electronic restructuring effect, facilitating charges transfer to Zn–S redox intermediates via the Zn─I─C5 σ‐coordinate bond. As summarized in Figure 3j, ZS‐CIM demonstrates a rapid and complete sulfur species conversion with anti‐corrosion of Zn anode, enhancing the CE of AZSBs.

### 2.4 Electrochemical Performances of AZSBs with CIM

**Figure 4:**
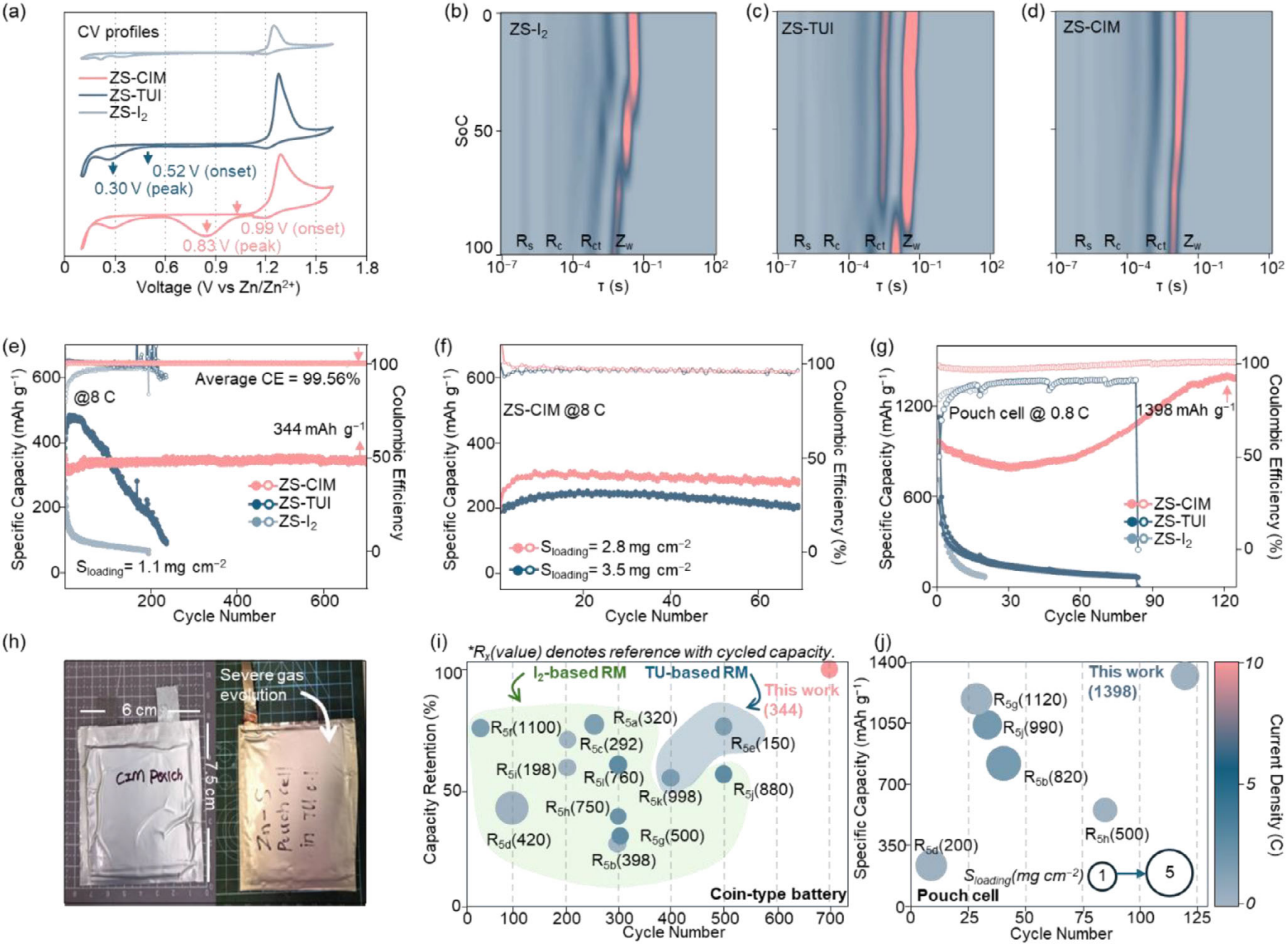
a) CV profiles of ZS‐I_2_, ZS‐TUI and ZS‐CIM under 0.5 mV s^−1^; b–d) In situ DRT profiles of ZS‐I_2_, ZS‐TUI and ZS‐CIM. e) Long‐term cycling of ZS‐I_2_, ZS‐TUI and ZS‐CIM at 8 C; f) Cycling performance tests of ZS‐CIM under various S_loading_ values of 2.8 mg cm^−2^ and 3.5 mg cm^−2^. g) Pouch cell tests of ZS‐I_2_, ZS‐TUI and ZS‐CIM at 0.8 C. h) Visual investigation of gas evolution in ZS‐CIM and ZS‐TUI. i–j) Comparison of electrochemical performance between reported AZSBs and ZS‐CIM in coin and pouch cells.

To validate the synergistic mechanism of CIM in AZSBs, a series of electrochemical tests including cyclic voltammetry (CV), in situ distribution of relaxation times (DRT), high‐ and low‐rate cycling tests were systematically conducted with coin and pouch cells. The CV profiles in **Figure** **4**a reveal that for ZS‐TUI, the S_8_ reduction reaction initiates at an onset potential of 0.52 V versus Zn/Zn^2+^, with its reduction peak appearing at 0.30 versus Zn/Zn^2+^ in ZS‐TUI. In contrast, ZS‐CIM exhibits a much higher onset potential for S_8_ reduction at 0.99 V versus Zn/Zn^2+^ than ZS‐TUI, with its corresponding reduction peak located at 0.83 V versus Zn/Zn^2+^. This demonstrates that ZS‐CIM exhibits reduced battery polarization compared to ZS‐TUI, which aligns well with its high discharge plateau as shown in Figure S18 (Supporting Information). In Figure 4b–d, the in situ DRT tests present that the charge transfer resistance (*R*_ct_) and Warburg diffusion (*Z*_w_) in the ZS‐CIM stay minimal and stable, demonstrating efficient charge transfer and ion diffusion. In contrast, both *R*_ct_ and *Z*_w_ increase in ZS‐I_2_ and ZS‐TUI, suggesting their sluggish electrochemical kinetics.

To evaluate CE and long‐term capacity retention of AZSBs, various coin cells with sulfur loading (S_loading_) of 1.1 mg cm^−2^ are tested at a high current density of 8 C, as shown in Figure 4e. The ZS‐I_2_ and ZS‐TUI suffer from low CEs mainly due to I_3_^−^‐mediated anode corrosion, as shown in Figure S19 (Supporting Information). As a result, the ZS‐I_2_ and ZS‐TUI fail after 195 and 236 cycles with capacity retentions of only 19% and 22%, respectively. In contrast, ZS‐CIM exhibits a remarkable average CE of 99.56%, achieving 344 mAh g^−1^ capacity after 700 cycles. Even with high S_loading_ of 2.8 mg cm^−2^ and 3.5 mg cm^−2^, as shown in Figure 4f, the ZS‐CIM exhibits high reversible capacity and excellent capacity retention. The capacity activation over the first few cycles is attributed to electrolyte infiltration and electrode wetting, which became more pronounced with higher S_loading_. This outstanding performance arises from the CIM's ability to synergistically enhance Zn–S redox kinetics while suppressing Zn anode corrosion, achieving high CE and long‐term capacity retention.

Evaluation of the performances in the pouch cell is conducted to evaluate the stability of ZS‐CIM. Specifically, cycling tests are conducted in pouch cells at 0.8 C (Figure 4g) and 0.07 C (Figure S20, Supporting Information). At 0.8 C, ZS‐I_2_ pouch cell exhibits rapid capacity decay, leading to complete failure after 20 cycles. Also, the capacity of ZS‐TUI pouch cell sharply dropped after 30 cycles, retaining only 13% of its initial capacity. In contrast, the ZS‐CIM pouch cell exhibits high cycling stability, maintaining a capacity of 1398 mAh g^−1^ after 120 cycles. Even at 0.07 C (Figure S20, Supporting Information), the ZS‐CIM pouch cell maintains high CEs. Notably, the phenomenon of capacity activation and the gradual improvement in sulfur utilization can also be observed in the ZS‐CIM pouch cell (Figure 4g). This phenomenon is investigated by SEM images of the cycled cathode surfaces in ZS‐TUI and ZS‐CIM pouch cells, as shown in Figure S21 (Supporting Information). The ZS‐TUI cathode shows a thick passivation layer of sulfur species, thus lowering the CE. In contrast, the ZS‐CIM cathode retains the porous morphology after cycling because of highly efficient Zn–S redox kinetics facilitated by CIM. Consistent with the observations in coin cells, Figure 4h further confirms that the ZS‐TUI pouch cell undergoes severe gas evolution. In contrast, the ZS‐CIM pouch cell shows good stability with no observable gas evolution. The performances of ZS‐CIM in both coin cells and pouch cells are compared with recently reported high‐performance AZSBs. As presented in Figure 4i,j and Table S11 (Supporting Information), ZS‐CIM achieved amongst the highest long‐term capacity retention in coin cells with a high average CE of 99.56%. This advantage is consistently maintained in pouch cells, further validating the synergistic mechanism of CIM in achieving a high capacity of 1398 mAh g^−1^ after 120 cycles.

## 3 Conclusion

In this work, a CIM catalyst with a stable covalent C5─I bond, and a dynamic electronic restructuring effect is reported. The synergistic mechanism enabled by CIM for high‐CE AZSBs can be summarized as follows: 1) the stable covalent C5─I bond firmly anchors iodine species to eliminate I_3_^−^‐mediated Zn anode corrosion; 2) the dynamic electronic restructuring effects enhance cathode kinetics by facilitating charge donation to Zn–S redox intermediates through the Zn─I─C5 coordination pathway. As a result, CIM‐based AZSBs achieve an outstanding CE of 99.56% and a capacity of 344 mAh g^−1^ after 700 cycles. The pouch cells employing CIM deliver a high capacity of 1398 mAh g^−1^ after 120 cycles. This rationally designed CIM presents a practical and scalable strategy for developing anti‐corrosive catalysts, providing critical insights into advancing CE and long‐term stability in aqueous metal‐sulfur battery technologies.

## Conflict of Interest

The authors declare no conflict of interest.

## Supporting information



Supporting Information

## Data Availability

The data that support the findings of this study are available from the corresponding author upon reasonable request.
