# Supplementary material for: Anti‐Corrosive Covalent Iodo‐Thiadiazole Catalyst Enables Aqueous Zn─S Batteries with High Coulombic Efficiency
Source: Adv Mater. 2025 Aug 18;37(43):e08570. doi: 10.1002/adma.202508570 (PMC12574616; doi:10.1002/adma.202508570)
Supplement: Supplementary file 1 — Supporting Information [file ADMA-37-e08570-s001.docx]

Supporting Information

for

Anti-Corrosive Covalent Iodo-Thiadiazole Catalyst Enables Aqueous Zn-S Batteries with High Coulombic Efficiency

Jiahao Liu, Yujie Chen, Han Wu, Chao Ye,* and Shi-Zhang Qiao*

J. Liu, Y. Chen, H. Wu, Dr. C. Ye, and Prof. S.-Z. Qiao

School of Chemical Engineering, The University of Adelaide, Adelaide, SA 5005, Australia

E-mail: [chao.ye@adelaide.edu.au](mailto:chao.ye@adelaide.edu.au); [s.qiao@adelaide.edu.au](mailto:s.qiao@adelaide.edu.au)

Experimental Section

**Chemical and materials**

2-amino-1,3,4-thiadiazole (C_2_H_3_N_3_S, powder, 97%), thiourea (NH_2_CSNH_2_, powder, ≥ 99.0%), and sulfur (S, powder, 99.98%) were purchased by Sigma-Aldrich. Zinc sulfate hexahydrate (ZnSO_4_∙6H_2_O, powder, ≥ 99.0%) was purchased by Chem-Supply. Carbon nanotube (CNT, in N-Methyl-2-pyrrolidone solutions, 0.4 wt.%) was purchased by Xianfeng Nano Co., Ltd.

**Synthesis of covalent iodo-thiadiazole redox mediator (CIM) and thiourea–iodide (TUI)**

For synthesis of CIM, commercially purchased 2-amino-1,3,4-thiadiazole and iodine (I_2_) were mixed with a molar ratio of 1:1 in dichloromethane and stirred at 0 ^o^C for 3 h at a stirring rate of 500 rpm. The obtained product was crystallized in an acetone solution and then dried in an oven at 60^o^C for 12 h to generate CIM powder. Firstly, commercially purchased thiourea and I_2_ were mixed with a molar ratio 1:1 in dichloromethane and stirred at 0 ^o^C for 3 h at a stirring rate of 500 rpm. The obtained product was dried in an oven at 60 ^o^C for 12 h to obtain TUI.

**Preparation of sulfur and carbon nanotube composite S@CNT**

The S and CNT powder was dispersed in an aqueous solution at a mass ratio of 1:1, stirred, and sonicated for 30 min. The resulting solution was transferred to an evaporation dish and dried in an oven at 80 ^o^C for 12 h, forming a thin gray film. The gray film was folded and wrapped with aluminum foil under an Ar atmosphere. It was subsequently transferred to a tube furnace under an Ar atmosphere and heated at a rate of 5 ^o^C min^−1^ to 155 ^o^C, held for 3 h to obtain S@CNT.

**Characterization methods**

In-situ synchrotron infrared micro spectroscopy was performed using the ATR mode of a Bruker VERTEX V70 spectrometer at the beamline of the ANSTO, covering the wavenumber range of 750-3900 cm^−1^. A CaF_2_ crystal and an open in-situ reaction cell were used, which was connected to a CHI 660E electrochemical workstation. The near-edge X-ray absorption fine structure (NEXAFS) data were collected by the soft X-ray beamline at the Australian Synchrotron. The in-situ inVia^TM^ confocal Raman test was combined with the CHI760E electrochemical station. An operando test model from GaossUnion was utilized to detect the electrolyte near the cathode interface from c.a. 100 cm^−1^ to 800 cm^−1^. A scanning electron microscope (SEM) and X-ray Energy Dispersive Spectroscopy (EDS) tests were implemented on an FEI Quanta 450 FEG device operating at 20 kV. X-ray photoelectron spectroscopy (XPS) test was performed on K-Alpha XPS under a pass energy of 30.0 eV using an Al K-Alpha gun. In the XPS fitting of S 2p3/2 and S 2p1/2 spectra, parameters were constrained based on spin-orbit splitting theory: intensity ratio fixed at 2:1, energy splitting at ~1.2 eV, consistent Gaussian-Lorentzian ratio, and identical the Full Width at Half Maximum (FWHM) for both peaks. The in-situ Distribution of Relaxation Times (DRT) was calculated using in-situ electrochemical Impedance Spectroscopy (EIS) data on the Biologic EC-Lab electrochemical station. The pH test of different electrolytes was performed using a Mettler Toledo FE20 suit at 20^o^C. The pH values of different electrolytes (2M ZnSO_4_, 2M ZnSO_4_ with 1 wt. % I_2_, 2M ZnSO_4_ with 1 wt. % TUI, and 2M ZnSO_4_ with 1 wt. % CIM) were measured using a Mettler Toledo FE20 instrument at 20^o^C, while their ionic conductivities were determined using an LC-DDB-1M conductivity meter under the same temperature condition.

**Electrochemical tests**

The 2M ZnSO_4_ electrolyte was used as the blank electrolyte. The addition of 1 wt.% I_2_ yielded the I_2_-modified electrolyte, 1 wt.% TUI yielded the TUI-modified electrolyte, and 1 wt.% CIM yielded the CIM-modified electrolyte. The S@CNT, Super P carbon, and PEFE were mixed at a mass ratio 7:2:1 to prepare the flexible electrode. The flexible electrode was uniformly coated onto a 100-mesh stainless steel mesh via roll pressing and dried in an oven at 60 ^o^C for 1 h. The obtained material was cut into 5 mm × 5 mm square electrode sheets and assembled into 2032-coin cells with a glass fiber separator and a zinc anode, using 100 µL of electrolyte. The sulfur loading ranged from 1 to 3.5 mg cm^−2^. For pouch cells, the dried stainless steel mesh electrode was prepared using the same process and cut into square sheets. A Zn-coated Cu foil was used as the anode, with a glass fiber separator. The cells were encapsulated using an aluminum-plastic composite film, the tabs were welded, and the cells were sealed using a vacuum heat sealer. The sulfur loading of pouch cell ranged from 1.3 to 1.8 mg cm^−2^. During the cycling and rate test, LAND CT2001 was performed from 0.05-1.60 V vs. Zn/Zn^2+^ at 0.07-8 C at 25 ^o^C, respectively. Cyclic voltammetry and chronoamperometry were performed using a Biologic Vmp-3e electrochemical workstation in a voltage range of 0.05-1.60 V vs. Zn/Zn^2+^.

**Supplemental Figures**


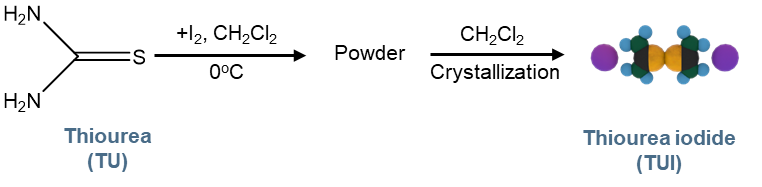


**Figure S1.** Iodination synthesis of TUI ionic complexes.

**
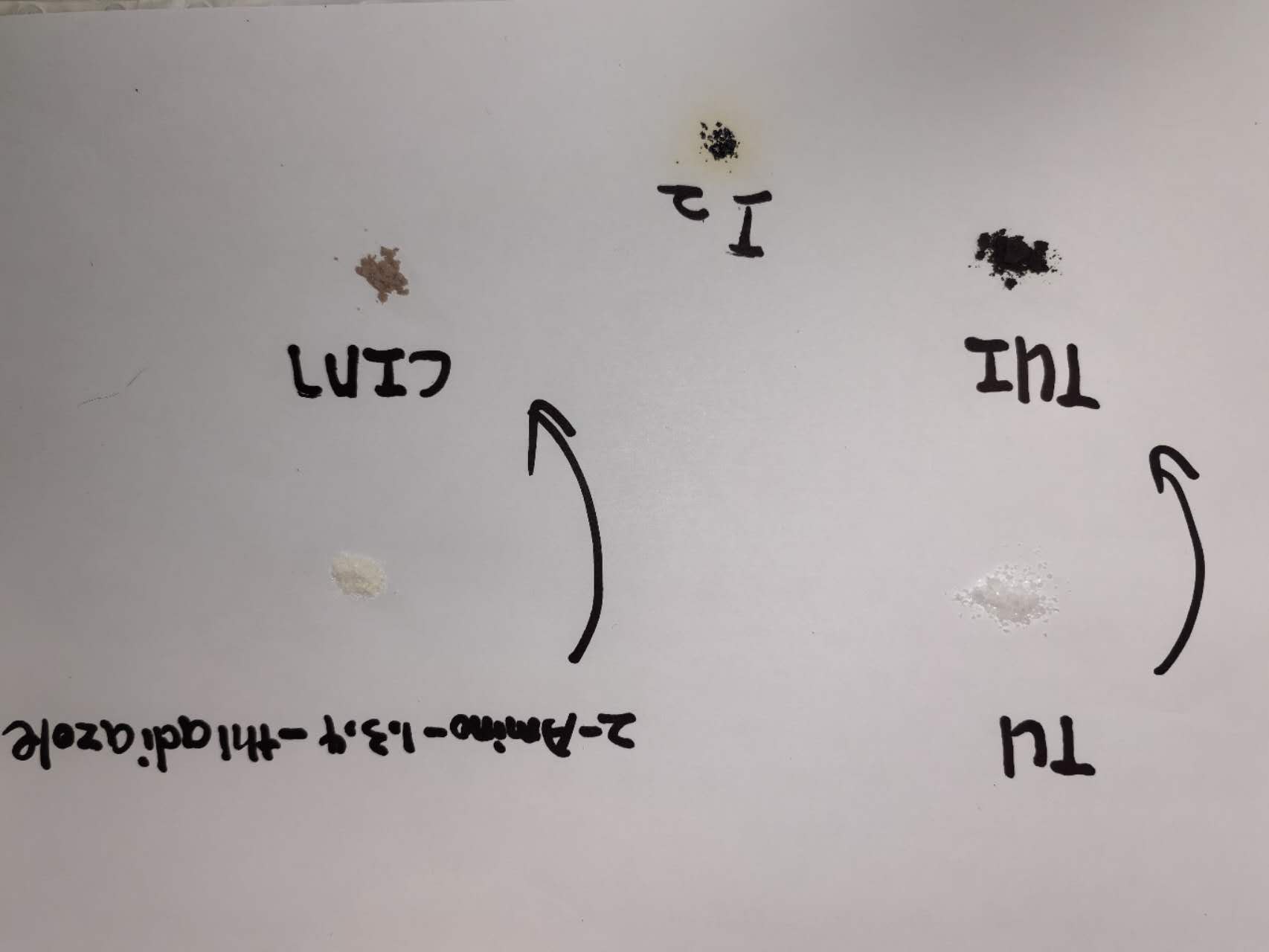
**

**Figure S2.** Image to show the different colors of the precursor of 2-AT and TU powders and the products of CIM and TUI powders.

**
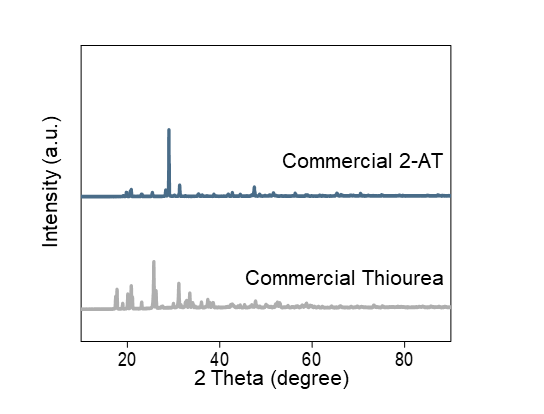
**

**Figure S3.** XRD patterns of precursor 2-AT and TU powders.

**
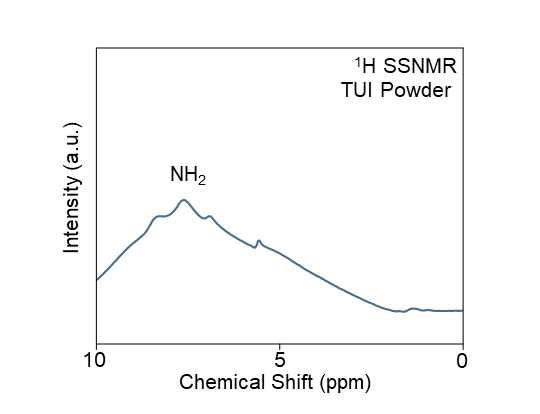
**

**Figure S4.** ^1^H SSNMR spectra of TUI powder (detailed spectral analysis in Table S8).

**
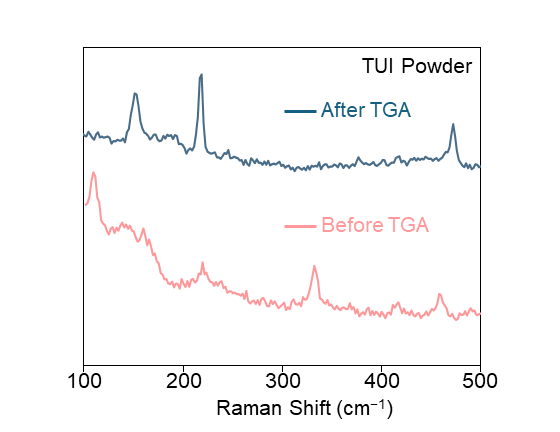
**

**Figure R5.** Raman spectra of TUI powders before and after TGA tests.


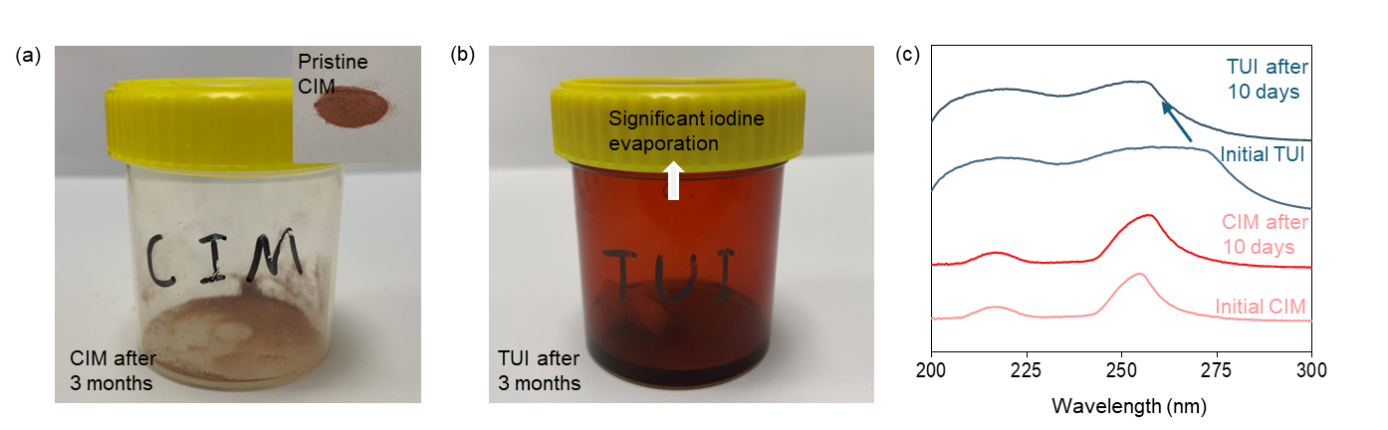


**Figure S6.** Air stability validation of TUI and CIM based on (a-b) visual observation after 3 months and (c) UV-vis test before and after 10 days.


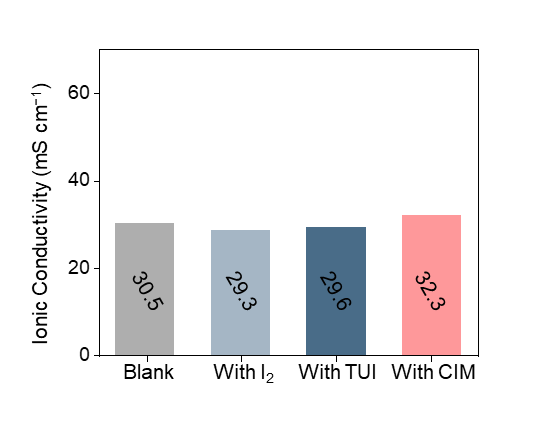


**Figure S7.** Conductivity of various electrolytes with 2M ZnSO_4_, ZZ-I_2_, ZZ-TUI, and ZZ-CIM.

**
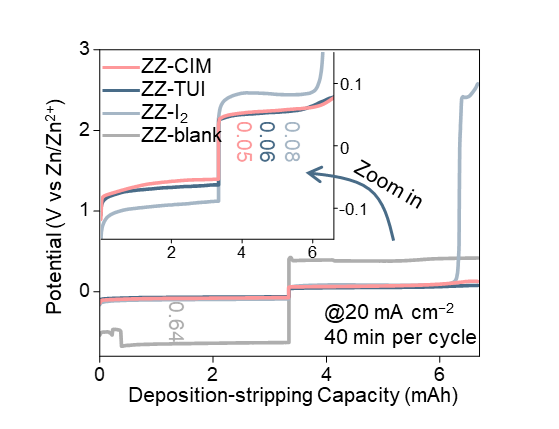
**

**Figure S8.** Polarization profiles of batteries with 2M ZnSO_4_, ZZ-I_2_, ZZ-TUI, and ZZ-CIM during the initial cycle.


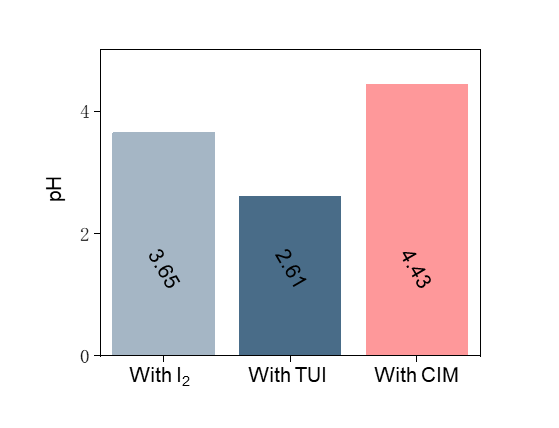


**Figure S9.** The pH values of electrolytes of 2M ZnSO_4_, ZZ-I_2_, ZZ-TUI, and ZZ-CIM.

**
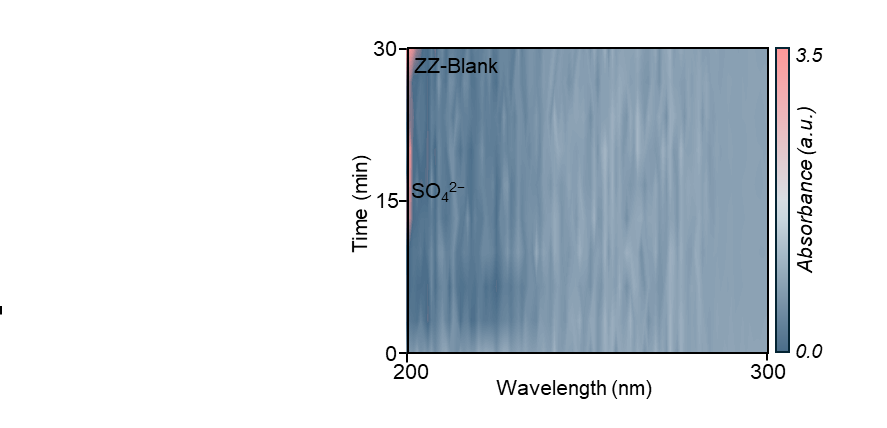
**

**Figure S10.** *In-situ* UV-Vis spectra of battery with 2M ZnSO_4_.


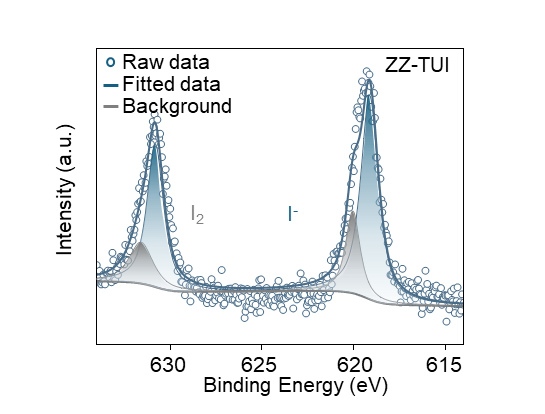


**Figure S11.** XPS analysis of the I 3d on Zn anodes with ZZ-TUI.


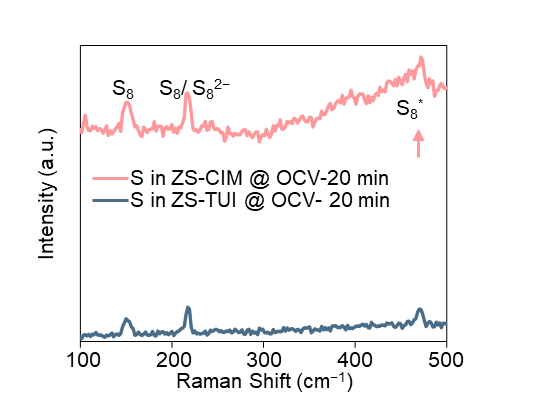


**Figure S12.** Raman spectrum of S in ZS-CIM and ZS-TUI batteries at open circuit voltage.

**
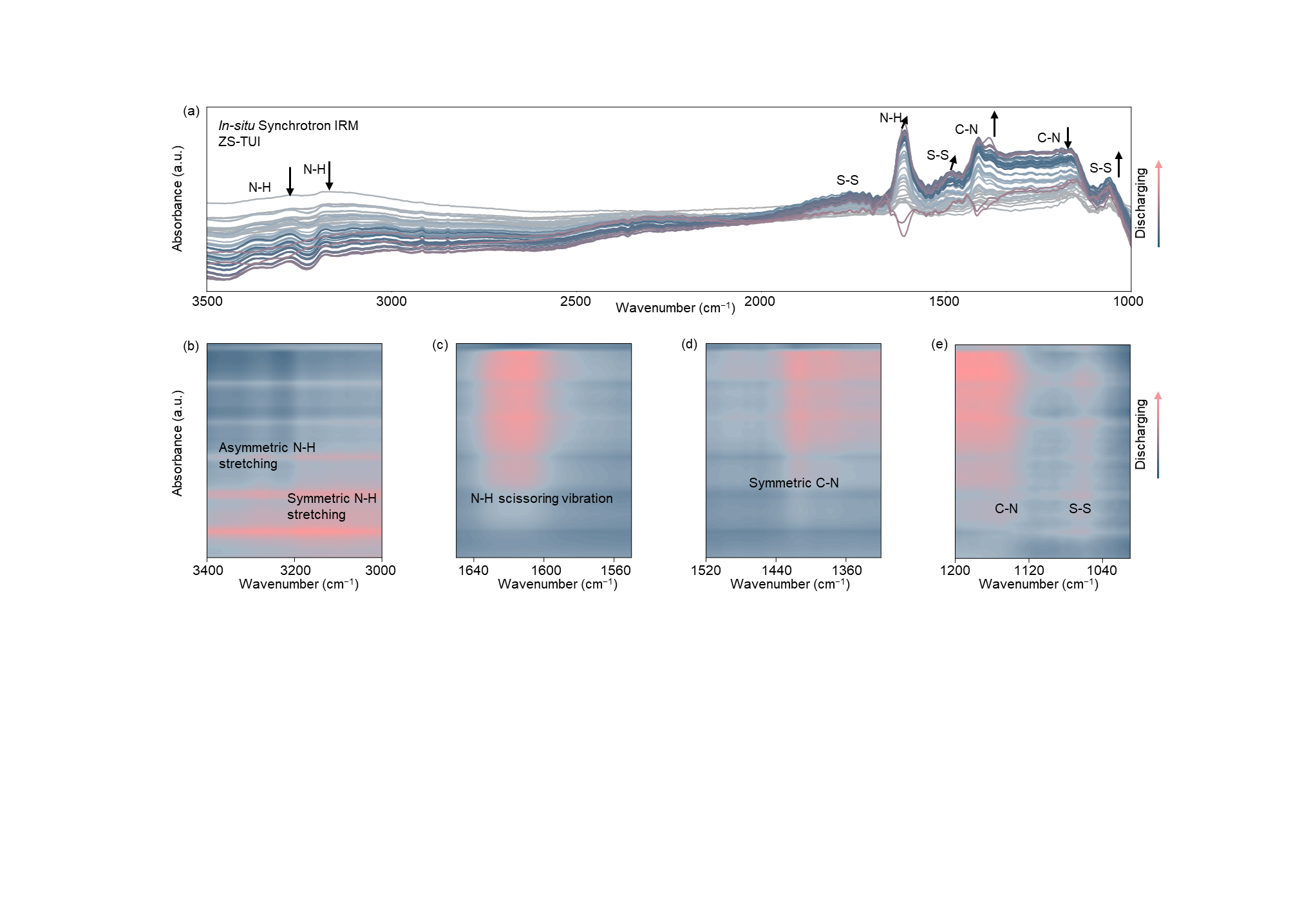
**

**Figure S13.** (a) *In-situ* synchrotron IRM analysis of ZS-TUI with (b-e) contour plots (detailed spectral analysis in Table S10).

**
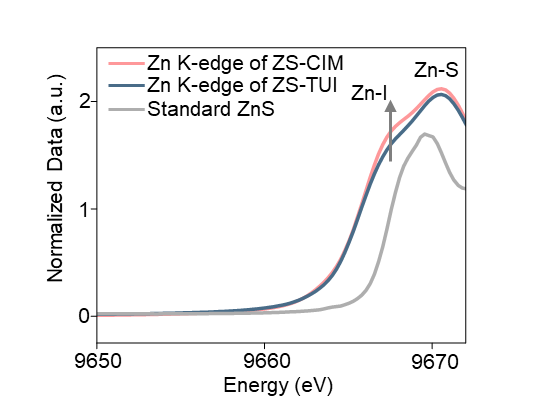
**

**Figure S14.** Synchrotron NEXAFS spectra of Zn K-edge signals of standard ZnS and the cathodes discharged at 0.7 V *vs.* Zn/Zn^2+^ in ZS-TUI and ZS-CIM.

**
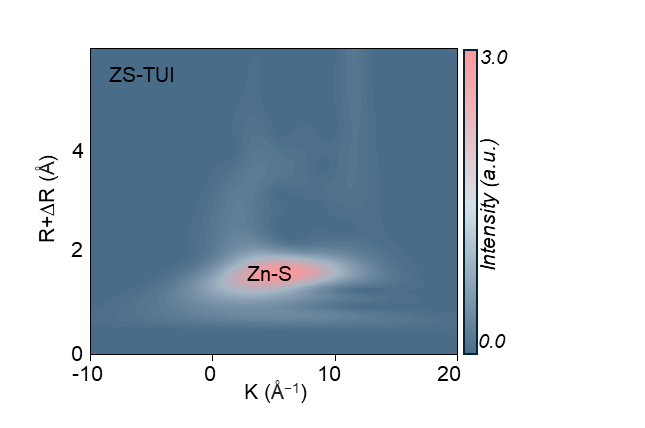
Figure S15.** X-ray absorption fine structure-based wavelet transform (WT-XAFS) analysis contour plots of cathode in ZS-TUI.

**
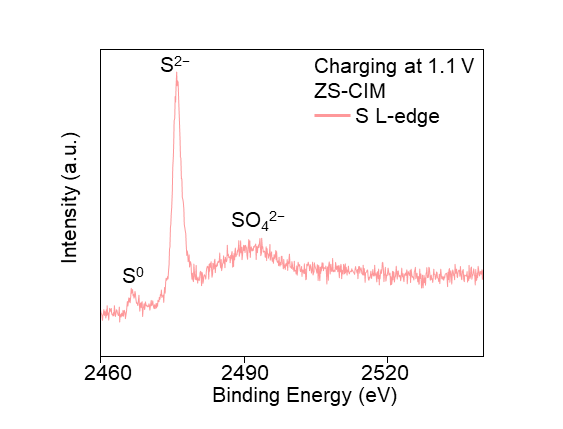
**

**Figure S16.** Synchrotron NEXAFS profiles of S L-edge signals in discharged ZS-CIM cathodes.

**
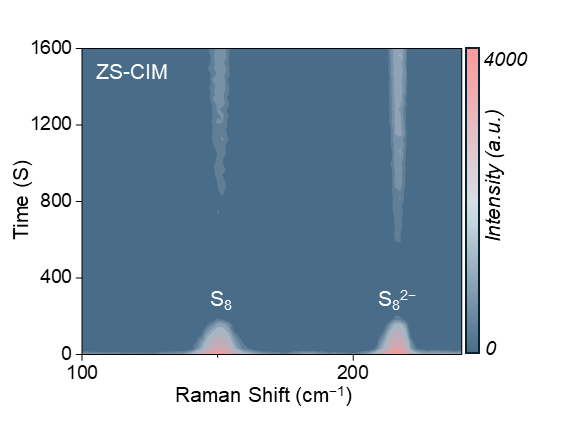
**

**Figure S17.** *In-situ* Raman spectra of ZS-CIM cathodes in one cycle.

**
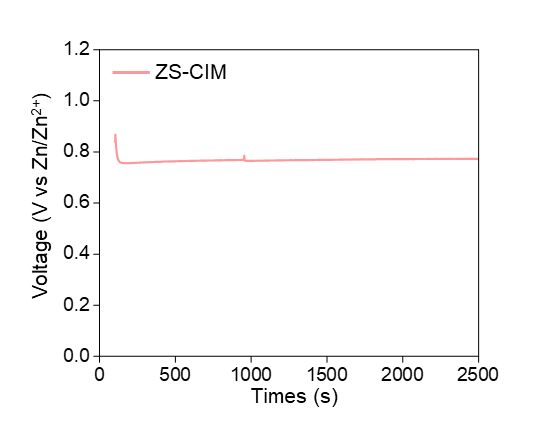
**

**Figure S18**. GCD profiles of ZS-CIM during discharge.


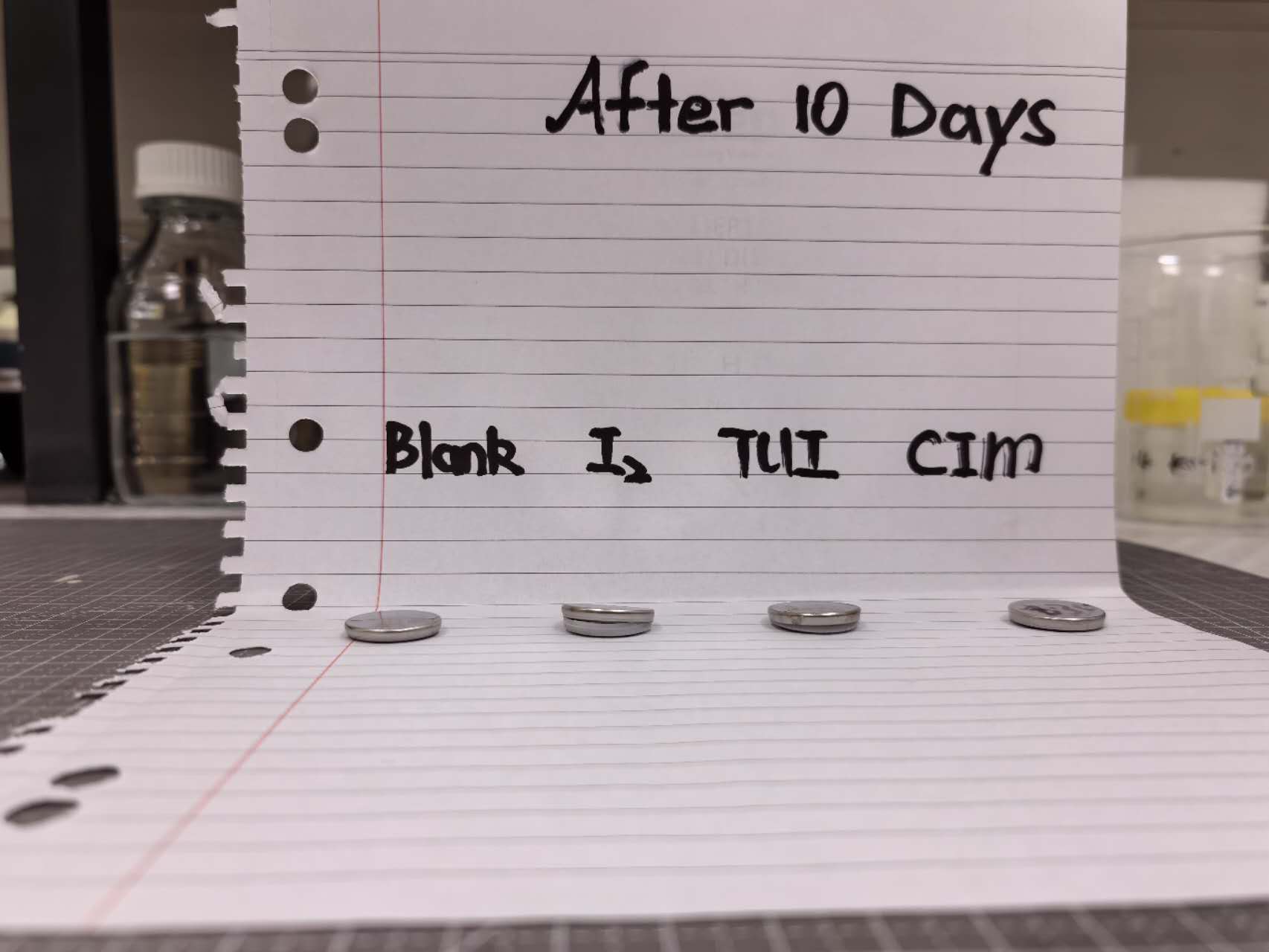


**Figure S19**. Investigation of OER-induced explosion in coin batteries of ZS-blank, ZS-I_2_, ZS-TUI, and ZS-CIM after 10-day cycling.


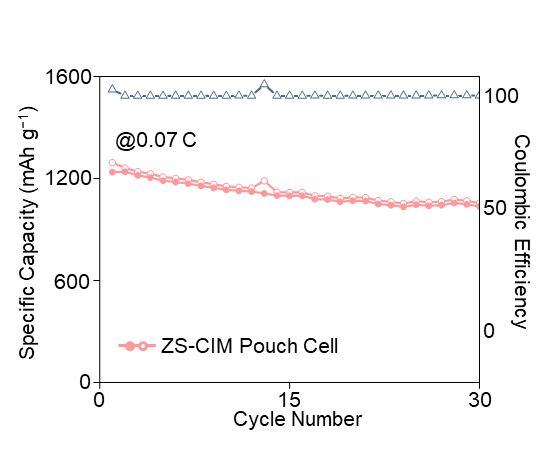


**Figure S20**. Low-rate stability test of the ZS-CIM pouch cell at 0.07 C.


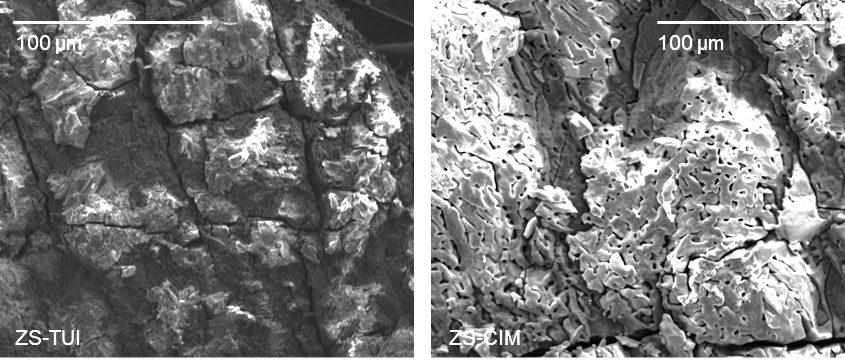


**Figure S21**. SEM images of ZS-TUI and ZS-CIM pouch cell cathodes after cycling.

**Supplemental Tables**

**Table S1.** FTIR spectral analysis of 2-AT powder.^[1]^

| **Wavenumber (cm^−1^)** | **Functional Group** | **Vibration Mode** |
| --- | --- | --- |
| 3272 | N-H Stretching | Amino group (-NH_2_). |
| 3074 | C-H Stretching | C-H stretching of the thiadiazole ring. |
| 1617 | C=N stretching | C=N stretching at position C2. |
| 1527 | C=N stretching | C=N stretching at position C5. |
| 1448 | C=N→C-N | Weak electron restructuring effect |
| 1022 | C-N Stretching | C-N stretching between the amino group and the ring. |
| 902 | C-H Bending | C-H bending of the thiadiazole ring. |
| 776 | C-S Stretching | C-S stretching in the thiadiazole ring. |
| 680 | Ring Vibration | Skeletal vibration of the thiadiazole ring. |

**Table S2.** Synchrotron IRM spectral analysis of CIM powder.^[2]^

| **Wavenumber (cm^−1^)** | **CIM Peak Assignment** | **Difference from 2-AT** |
| --- | --- | --- |
| 3272 | N-H Stretching | Retained from 2-AT. |
| 3159 | N-H Stretching (split) | A split N-H bond caused by the enhanced Electron restructuring. |
| 3074 | C-H Stretching | Retained from 2-AT. |
| 1617 | C=N Stretching (C2) | Retained from 2-AT. |
| 1599 | C=N Stretching (C2) | Shifted from 1527 cm^−1^ due to the C5-I bond-induced electron localization in the C2=N bond. |
| 1401 | Enhanced C=N→C-N | Shifted from 1448 cm^−1^, indicating an increased contribution of C-N character. |
| 1022 | C-N Stretching | Retained from 2-AT. |
| 902 | C-H Bending | Retained from 2-AT. |
| 776 | C-S Stretching | Retained from 2-AT. |
| 662 | Ring Vibration | Shifted from 680 cm^−1^ due to C-I bond. |
| 596 | C-I Stretching | A new peak, C-I bond formation. |
| 549 | C-I Bending and Ring Vibration | A new peak, mixed by C-I bending and ring vibrations |

**Table S3.** Raman spectral analysis of 2-AT powder.^[3]^

| **Raman Shift (cm^−1^)** | **Vibrational Mode** | **Explanation** |
| --- | --- | --- |
| 1226 | Ring Breathing Mode | Reflects the symmetric expansion/contraction of the thiadiazole ring, consistent with C-N/C-S coupling. |
| 1145 | C-N Stretching (at the C2 position) | Stretching vibration of the C2-N bond between the amino group and the ring. |
| 885 | Ring skeletal vibration | Likely a ring vibration mode rather than C-S stretching. |
| 771 | Ring skeletal vibration (C-S/N-C coupled) | Consistent with the thiadiazole ring’s framework vibrations. |
| 686 | Ring out-of-plane bending | Out-of-plane ring deformation |
| 581 | C-S bending | Assignable to C-S bending or ring distortion |
| 402 | Ring twisting | Likely reflects low-frequency ring-twisting |

**Table S4.** Raman spectral analysis of CIM powder.^[4]^

| **Raman Shift (cm^−1^)** | **Vibrational Mode** | **Explanation** |
| --- | --- | --- |
| 1368 | Enhanced Electron restructuring | Reflects increased C-N character due to the C-I bond’s electron-withdrawing effect. |
| 1226 | Ring Breathing Mode | Retained from 2-AT. |
| 1145 | C2-N Stretching | Retained from 2-AT. |
| 1108 | Ring vibration coupled with the C-I effect | Likely a ring vibration mode influenced by the C-I bond’s electronic perturbation |
| 885 | Ring skeletal vibration | Retained from 2-AT. |
| 771 | Ring skeletal vibration (C-S/N-C coupled) | Retained from 2-AT. |
| 686 | Ring out-of-plane bending | Retained from 2-AT. |
| 581 | C-S bending | Retained from 2-AT. |
| 402 | Ring Twisting | Retained from 2-AT. |
| 329 | C-I Bending or iodine-lattice coupling | Weak C-I bending or iodine-lattice coupling |
| 227 | Ring Twisting with C-I Influence | Lattice vibrations influenced by the heavy iodine atom |
| 110 | Molecular or Lattice Vibration | Intermolecular interactions. |

**Table S5.** Raman spectral analysis of TUI powder.^[4-5]^

| **Raman Shift (cm^−1^)** | **Vibration Mode** | **Explanation** |
| --- | --- | --- |
| 655 | C-S stretching | Stretching vibration of the C-S bond |
| 568 | S-S stretching | Consistent with symmetric S-S stretching |
| 458 | N-C-N bending | Bending vibration of the N-C-N group |
| 424 | C-S bending | Bending vibration of the C-S bond |
| 333 | Lattice vibration | Lattice distortions involving iodide ions |
| 221 | Lattice vibration | A collective lattice mode influenced by iodide’s mass |
| 157 | I^−^  lattice vibration | Consistent with low-frequency lattice vibrations of iodide ions in the crystalline framework. |

**Table S6.** ^1^H SSNMR spectral analysis of CIM powder. ^[1a]^

| **Assignment** | **Chemical Shift** | **Explanation** |
| --- | --- | --- |
| N-H (Adjacent to C-I) | 9.2 ppm | The N-H proton adjacent to the C-I bond experiences deshielding due to the strong electron-withdrawing effect of iodine, shifting downfield. |
| N-H (Remote from C-I) | 8.2 ppm | The N-H proton remote from the C-I bond may experience increased shielding due to electronic redistribution or weakened hydrogen bonding, shifting upfield. |
| C-H (Thiadiazole Ring) | 6.9 ppm | The C-H proton on the ring may experience increased shielding due to the overall electron density redistribution caused by the C-I bond despite its electron-withdrawing effect. |

**Table S7.** ^13^C SSNMR spectral analysis of CIM powder. ^[1a]^

| **Assignment** | **Chemical Shift** | **Explanation** |
| --- | --- | --- |
| C2 (C=N in Thiadiazole Ring) | 180.3 ppm | Carbon at position 2 (C2), part of the C=N double bond in the thiadiazole ring. Highly deshielded due to conjugation and the electron-withdrawing effect of sulfur. |
| C5 (C-I Bond) | 165.3 ppm | Carbon at position 5 (C5), substituted with iodine. The strong electron-withdrawing effect (-I) of iodine causes deshielding, but conjugation and solid-state anisotropy partially offset this effect. |
| Partial C5-N | 155.5 ppm | Likely arises from splitting due to the dynamic equilibrium between C=N and C-N configurations or crystal packing effects in the solid state. |

**Table S8.** ^1^H SSNMR spectral analysis of TUI powder.^[6]^

| **Chemical Shift (ppm)** | **Assignment** |
| --- | --- |
| 7.61 (Main Peak) | Symmetric NH_2_ groups interacting with S-S bonds; |
| 8.49 (Major Side Peak) | NH_2_ groups near I^−^ experience enhanced ionic interactions; |
| 6.82 (Secondary Peak) | NH_2_ groups with weaker hydrogen bonding interactions, likely influenced by the S-S bond polarity. |
| 5.59 (Minor Side Peak) | NH_2_ groups in weaker electronic environments, possibly due to local hydrogen bond disruption. |

**Table S9.** *In-situ* synchrotron IRM spectral analysts of the ZS-CIM. ^[1-2]^

| **Wavenumber (cm^−1^)** | **Shift Trend** | **Assignment and Explanation** |
| --- | --- | --- |
| 3297 | Amplified | Amino free stretching vibration on C2, amplified by the long-range electron delocalization of the thiadiazole ring due to the electron restructuring effect. |
| 3115 | Amplified | The formation of split N4-H induced by the electron restructuring effect |
| 3034 | Amplified | C-H of the thiadiazole ring. |
| 1635 | Amplified | C2=N stretching vibration, amplified by the long-range electron delocalization of the thiadiazole ring due to the electron restructuring effect. |
| 1509 | Amplified | Dynamic reduction of the electron restructuring effect. |
| 1420 | Amplified and redshifted to 1410 | Amplified electron restructuring effect. |
| 1069 | Amplified | C2-N of the amino group. |

**Table S10.** *In-situ* synchrotron IRM spectral analysts of the ZS-TUI. ^[1-2, 7]^

| **Wavenumber (cm^−1^)** | **Intensity Change** | **Shift Trend** | **Functional Group or Bond** |
| --- | --- | --- | --- |
| 3282 | Decrease | No significant shift | Free stretching of NH_2_. |
| 3181 | Decrease | No significant shift | Reduced H-bonding in N-H by I^−^ release. |
| 1754 | Increase | No significant shift | C=O vibration. |
| 1619 | Increase | Redshift to 1611 | NH_2_ electronic perturbation by I^−^ release. |
| 1500 | Increase | Redshift to 1489 | Structural rearrangement of TUI |
| 1411 | Increase | No significant shift | Enhanced C-N stretching polarization. |
| 1154 | Decrease | Blueshift to 1160 | Weakened C-N bonding due to electron loss. |
| 1059 | Increase, significant growth | C-S stretching | S-S bond cleavage → C-S bond stabilization. |

**Table S11.** Electrochemical performances of reported coin and pouch cell AZSBs.

| **Ref** | **RMs/ Catalysts** | **S_loading_**  mg cm^⁻2^ | **High-rate metrics**  mAh g^⁻1^ | **Low-rate metrics**  mAh g^⁻1^ | **Pouch cell metrics** mAh g^⁻1^ |
| --- | --- | --- | --- | --- | --- |
| 1^[8]^ | Me_3_PhN^+^Cl^−^ | 5.1 | 420 (@3Ag^-1^ after 100 cycles), CR=45%^*^ |  | 200 (@0.1C after 20 cycles) |
| 2^[9]^ | N−CNF | 1.0 | 750 (@4Ag^-1^ after 300 cycles), CR=43% |  | 500 (@1Ag^-1^ after 100cycles) |
| 3^[10]^ | Ti_3_C_2_T_x_ | 2 |  | 292 (@0.5Ag^-1^ after 300 cycles), CR=76% |  |
| 4^[11]^ | I_2_ | 2.0 | 398 (@2C after 300 cycles), CR=35% | 780 (@0.5C after 50 cycles), CR=51% | 820 (@1C after 40 cycles) |
| 5^[12]^ | TU | 1.0 | 760 (@5Ag^-1^ after 300 cycles), CR=57% | 200 (@1Ag^-1^ after 20 cycles), CR=13% |  |
| 6^[13]^ | Br_2_ | 1.8 | 998 (@3Ag^-1^ after 400 cycles), CR=52% | 1400 (@0.5Ag^-1^ after 50 cycles), CR=86% |  |
| 7^[14]^ | I_2_ | 1.2 | 880 (@4Ag^-1^ after 500 cycles), CR=54% | 900 (@0.5Ag^-1^ after 50 cycles), CR=66% | 990 (@2Ag^-1^ after 200 cycles) |
| 8^[15]^ | I_2_ | 1.0 | 198 (@1Ag^-1^ after 200 cycles), CR=44% | 510 (@0.5Ag^-1^ after 30 cycles), CR=63% |  |
| 9^[16]^ | ZnI_2_+TU | 2.5 | 500 (@2Ag^-1^ after 300 cycles), CR=45% | 1070 (@0.5Ag^-1^ after 50 cycles), CR=77% | 1120(@0.2Ag^-1^ after 35cycles) |
| 10^[17]^ | ZnClO_4_ | 2 | 150 (@2Ag^-1^ after 500 cycles), CR=78% |  |  |
| 11^[18]^ | ZnI_2_ | 1 |  | 1100 (@1Ag^-1^ after 50 cycles), CR=67% |  |
| 12^[19]^ | ZnI_2_ | 1.7 | 320 (@3Ag^-1^ after 250 cycles), CR=71% | 920 (@1Ag^-1^ after 50 cycles), CR=68% |  |
| This work | CIM | 1.1-3.5 | 344 (@8C after 700 cycles), CE=99.56%; CR=104.5% | 1398 (@0.8C after 120 cycles) | 1398 (@0.8C after 120 cycles) |

^*^CR stands for capacity retention rate after cycling.

**References**

[1] a) M. Er, G. Isildak, H. Tahtaci, T. Karakurt, *J. Mol. Struct.* **2016**, *1110*, 102-113; b) M. Zhang, H. Yin, L. Wan, H. Gao, S. Liu, Y. Liu, *Carbon Lett.* **2024**, *34*, 1155-1164.

[2] M. Karabacak, D. Karagöz, M. Kurt, *J. Mol. Struct.* **2008**, *892*, 25-31.

[3] a) T. A. Mohamed, A. E. Hassan, I. A. Shaaban, A. M. Abuelela, W. M. Zoghaib, *J. Mol. Struct.* **2017**, *1130*, 434-441; b) I. A. Shaaban, A. E. Hassan, A. M. Abuelela, W. M. Zoghaieb, T. A. Mohamed, *J. Mol. Struct.* **2016**, *1103*, 70-81.

[4] E. Loh, *J. Raman Spectrosc.* **1975**, *3*, 327-333.

[5] R. G. Kumari, V. Ramakrishnan, M. L. Carolin, J. Kumar, A. Sarua, M. Kuball, *Spectrochim. Acta-A* **2009**, *73*, 263-267.

[6] M. Chayah, M. E. Camacho, M. D. Carrión, M. A. Gallo, *Magn. Reson. Chem.* **2016**, *54*, 793-799.

[7] K. Kargosha, M. Khanmohammadi, M. Ghadiri, *Anal. Chim. Acta* **2001**, *437*, 139-143.

[8] W. Wu, S. Wang, L. Lin, H.-Y. Shi, X. Sun, *Energy Environ. Sci.* **2023**, *16*, 4326-4333.

[9] J. Li, J. Liu, F. Xie, R. Bi, L. Zhang, *Angew. Chem. Int. Ed.* **2024**, *63*, e202406126.

[10] K. K. Sonigara, J. V. Vaghasiya, C. C. Mayorga-Martinez, M. Pumera, *npj 2D Mater. Appl.* **2023**, *7*, 45.

[11] J. Li, Z. Cheng, Z. Li, Y. Huang, *Mater. Horiz.* **2023**, *10*, 2436-2444.

[12] G. Chang, J. Liu, Y. Hao, C. Huang, Y. Yang, Y. Qian, X. Chen, Q. Tang, A. Hu, *Chem. Eng. J.* **2023**, *457*, 141083.

[13] S. Wang, W. Wu, Q. Jiang, C. Li, H.-Y. Shi, X.-X. Liu, X. Sun, *Chem. Sci.* **2025**, *16*, 1802-1808.

[14] H. Zhang, M. Yang, J. Xiao, Z. Wu, W. Xin, X. Xiao, M. Niu, Z. Yan, Z. Zhu, *Adv. Funct. Mater.* **2024**, *34*, 2406125.

[15] D. Patel, A. Dharmesh, Y. Sharma, P. Rani, A. K. Sharma, *Chem. Engi. J.* **2024**, *479*, 147722.

[16] J. Li, J. Cong, Y. Ren, H. Ji, Z. Li, Y. Huang, *Energy Storage Mater.* **2024**, *70*, 103541.

[17] Z. Chen, Z. Huang, J. Zhu, D. Li, A. Chen, Z. Wei, Y. Wang, N. Li, C. Zhi, *Adv. Mater.* **2024**, *36*, 2402898.

[18] P. Hei, Y. Sai, C. Liu, W. Li, J. Wang, X. Sun, Y. Song, X.-X. Liu, *Angew. Chem. Int. Ed.* **2024**, *63*, e202316082.

[19] Y. Guo, R. Chua, Y. Chen, Y. Cai, E. J. J. Tang, J. J. N. Lim, T. H. Tran, V. Verma, M. W. Wong, M. Srinivasan, *Small* **2023**, *19*, 2207133.
